# GDNF Secreting Human Neural Progenitor Cells Protect Dying Motor Neurons, but Not Their Projection to Muscle, in a Rat Model of Familial ALS

**DOI:** 10.1371/journal.pone.0000689

**Published:** 2007-08-01

**Authors:** Masatoshi Suzuki, Jacalyn McHugh, Craig Tork, Brandon Shelley, Sandra M. Klein, Patrick Aebischer, Clive N. Svendsen

**Affiliations:** 1 The Waisman Center, University of Wisconsin-Madison, Madison, Wisconsin, United States of America; 2 Departments of Anatomy and Neurology, University of Wisconsin-Madison, Madison, Wisconsin, United States of America; 3 Brain & Mind Institute, Ecole Polytechnique Fédérale de Lausanne (EPFL), Lausanne, Switzerland; The Jackson Laboratory, United States of America

## Abstract

**Background:**

Amyotrophic lateral sclerosis (ALS) is a fatal, progressive neurodegenerative disease characterized by rapid loss of muscle control and eventual paralysis due to the death of large motor neurons in the brain and spinal cord. Growth factors such as glial cell line derived neurotrophic factor (GDNF) are known to protect motor neurons from damage in a range of models. However, penetrance through the blood brain barrier and delivery to the spinal cord remains a serious challenge. Although there may be a primary dysfunction in the motor neuron itself, there is also increasing evidence that excitotoxicity due to glial dysfunction plays a crucial role in disease progression. Clearly it would be of great interest if wild type glial cells could ameliorate motor neuron loss in these models, perhaps in combination with the release of growth factors such as GDNF.

**Methodology/Principal Findings:**

Human neural progenitor cells can be expanded in culture for long periods and survive transplantation into the adult rodent central nervous system, in some cases making large numbers of GFAP positive astrocytes. They can also be genetically modified to release GDNF (hNPC^GDNF^) and thus act as long-term ‘mini pumps’ in specific regions of the rodent and primate brain. In the current study we genetically modified human neural stem cells to release GDNF and transplanted them into the spinal cord of rats over-expressing mutant SOD1 (SOD1^G93A^). Following unilateral transplantation into the spinal cord of SOD1^G93A^ rats there was robust cellular migration into degenerating areas, efficient delivery of GDNF and remarkable preservation of motor neurons at early and end stages of the disease within chimeric regions. The progenitors retained immature markers, and those not secreting GDNF had no effect on motor neuron survival. Interestingly, this robust motor neuron survival was not accompanied by continued innervation of muscle end plates and thus resulted in no improvement in ipsilateral limb use.

**Conclusions/Significance:**

The potential to maintain dying motor neurons by delivering GDNF using neural progenitor cells represents a novel and powerful treatment strategy for ALS. While this approach represents a unique way to prevent motor neuron loss, our data also suggest that additional strategies may also be required for maintenance of neuromuscular connections and full functional recovery. However, simply maintaining motor neurons in patients would be the first step of a therapeutic advance for this devastating and incurable disease, while future strategies focus on the maintenance of the neuromuscular junction.

## Introduction

Amyotrophic lateral sclerosis (ALS) is a fatal, progressive neurodegenerative disease characterized by rapid loss of muscle control and eventual paralysis due to the death of large motor neurons in the spinal cord and brainstem [1], [2]. The cause of sporadic ALS remains unclear. However, in a small group of familial ALS (FALS) patients, a clear genetic link to point mutations in superoxide dismutase 1 (SOD1) has been shown [3]. This has led to the generation of transgenic mice and rats over-expressing multiple copies of mutant SOD1 (SOD1^G93A^) that have many of the characteristics of both the familial and sporadic form of human disease [4], [5].

Motor neuron death in ALS is a complex process, and may involve multiple pathways including formation of protein aggregates, axonal transport defects, oxidative damage, mitochondrial defects, and alterations in calcium homeostasis [6]. Healthy motor neurons express components of glial cell line derived neurotrophic factor (GDNF) receptors and can bind, internalize and transport the protein in both retro- and anterograde directions [7], [8]. Many studies have shown that GDNF protects motor neurons from degeneration *in vitro* [9]. However, delivery of GDNF *in vivo* has been hindered by limited bio-availability, inability of this protein to cross the blood-brain barrier or penetrate gray matter, and by a relatively short half-life. Gene therapy approaches using adenoviral vectors encoding GDNF or cells releasing GDNF have shown that muscle delivery of this factor and subsequent retrograde transport to motor neurons in the spinal cord can be effective in slowing degeneration in the mouse SOD 1 mutant [10]–[12]. However, retrograde transport may be affected early in the disease [13] and larger animals such as rats and monkeys may be less efficient at taking back the protein to the spinal cord thus limiting this approach in patients. Only one study has attempted direct delivery of GDNF to the motor neurons of the SOD1^G93A^ mouse lumbar spinal cord using lentiviral vectors, and this did not result in lumbar motor neuron protection, although there was some modest protection of facial motor neurons [14]. Furthermore, recent evidence from transgenic mice suggests that direct expression of GDNF by astrocytes within the spinal cord does not result in motor neuron protection when compared to muscle delivery [15]. However, in both of these studies, sick astrocytes and/or neurons carrying the SOD1 mutation were forced to release GDNF which may have compromised the effectiveness of this approach.

Although there may be a primary dysfunction in the motor neuron itself, there is also increasing evidence that excitotoxicity due to astrocyte dysfunction and inflammatory processes from microglia activation play a crucial role in disease progression [2], [4], [16]–[18]. Most of the studies to date have focused on modulating the ratio of mutant SOD1 over expression and normal cells in transgenic mice and have shown that reducing the number of glia expressing the mutation can have a significant impact on motor neuron survival and disease progression [17]–[20]. While of great interest, these studies did not assess whether adding wild type glial cells into an environment where every cell carries the mutation would have a similar effect, or whether growth factors may further ameliorate motor neuron death. Results from this experiment would have clear clinical implications for patients with the disease.

Human neural progenitor cells (hNPC) can be expanded in culture for long periods and survive transplantation into the adult rodent central nervous system, in some cases making large numbers of GFAP positive astrocytes at longer survival times [21]–[26]. These cells can also be genetically modified to release GDNF (hNPC^GDNF^) and thus act as long-term ‘mini pumps’ in specific regions of the rodent and primate brain [27]. We have also shown that hNPC^GDNF^ can survive transplantation into the lumbar spinal cord of the SOD1^G93A^ rat and continue to release GDNF within this degenerative environment [28]. In the current study we show that delivery of GDNF by these cells has a remarkable protective effect on motor neuron cell death in highly chimeric regions of the SOD1^G93A^ rat spinal cord, even at disease end point. This was not through modulation of the host glial response, or up regulation of glutamate transporters such as glutamate transporter-1 (GLT1) around the surviving motor neurons. Surprisingly, these surviving motor neurons did not appear to maintain their contact with muscle and there were no improvements in ipsilateral hind limb function. The potential to maintain dying motor neurons with GDNF in this severe degenerative model points to possible novel stem cell treatment strategies for ALS.

## Methods

### Animals

Breeder SOD1^G93A^ rats, which had been originally generated by Howland *et al.* [5], were obtained from Taconic (http://www.taconic.com/), and colonies were developed by crossing male founders with female Taconic Sprague Dawley rats (http://www.taconic.com/). We began breeding the SOD1^G93A^ rat colony from founder animals and found a constant but predictable drift in disease onset and duration in addition to a gender specific effect with males being more severely affected than females [29]. This is entirely consistent with the pattern of familial ALS in humans [1], [2]. We used two different cohorts of rats and the variation with regard to disease onset and progression in our hSOD1^G93A^ rats was observed as previously described [29]. Heterozygous SOD1^G93A^ progeny were identified with polymerase chain reaction (PCR) of tail DNA with primers specific for hSOD1. They were maintained in a room with controlled illumination (lights on 0500–1900 h) and temperature (23±1°C), and given free access to laboratory chow and tap water. Rats were considered end-stage (endpoint) when they no longer exhibited reflexes allowing them to right themselves within 30 seconds. Body weight measurement and all behavioral testing began at an age of 65 days and continued until endpoint. All the animal procedures in the present study were carried out in accordance with the guidelines for University of Wisconsin-Madison and National Institutes of Health standards of animal care.

### Cell culture

Human fetal tissue (between 10 and 15 weeks of conception) was obtained from the Birth Defects Laboratory at the University of Washington. The method of collection conformed to the guidelines recommended by National Institutes of Health for the collection of such tissues and set out by the University of Washington and the University of Wisconsin, Madison. Institutional Review board approval was obtained for all of these studies. Human cortical neural progenitor/stem cells (M031 line) were prepared from fetal brains and induced to proliferate as neurospheres using established passaging methods to achieve optimal cellular expansion as previously described in detail [24]. Freshly dissected fetal tissue was dissociated in 0.1% trypsin and seeded into T75 flasks at a density of 200,000 cells per ml of maintenance medium [Dulbecco's modified Eagle medium (DMEM)/Ham's F12 (7∶3) containing penicillin/streptomycin/amphotericin B (PSA, 1% v/v)] supplemented with B27 (2% v/v; Invitrogen, http://probes.invitrogen.com/), EGF (20 ng/ml; Sigma-Aldrich, http://www.sigmaaldrich.com/), and fibroblast growth factor-2 (FGF-2; 20 ng/ml; R&D Systems, http://www.rndsystems.com/) with heparin (5 µg/ml; Sigma). Neurosphere colonies rapidly formed and were passaged every 14 days by sectioning neurospheres into 200 µm using an automated tissue chopper as described previously [24]. At 2 weeks after the first passage, the cells were switched to maintenance medium containing N2 supplement (1%; Invitrogen) and 20 ng/ml EGF. After 10 weeks of growth, 10 ng/ml LIF (Chemicon, http://www.chemicon.com/) was added to enhance expansion rates.

### Lentiviral infection

**Figure 1:**
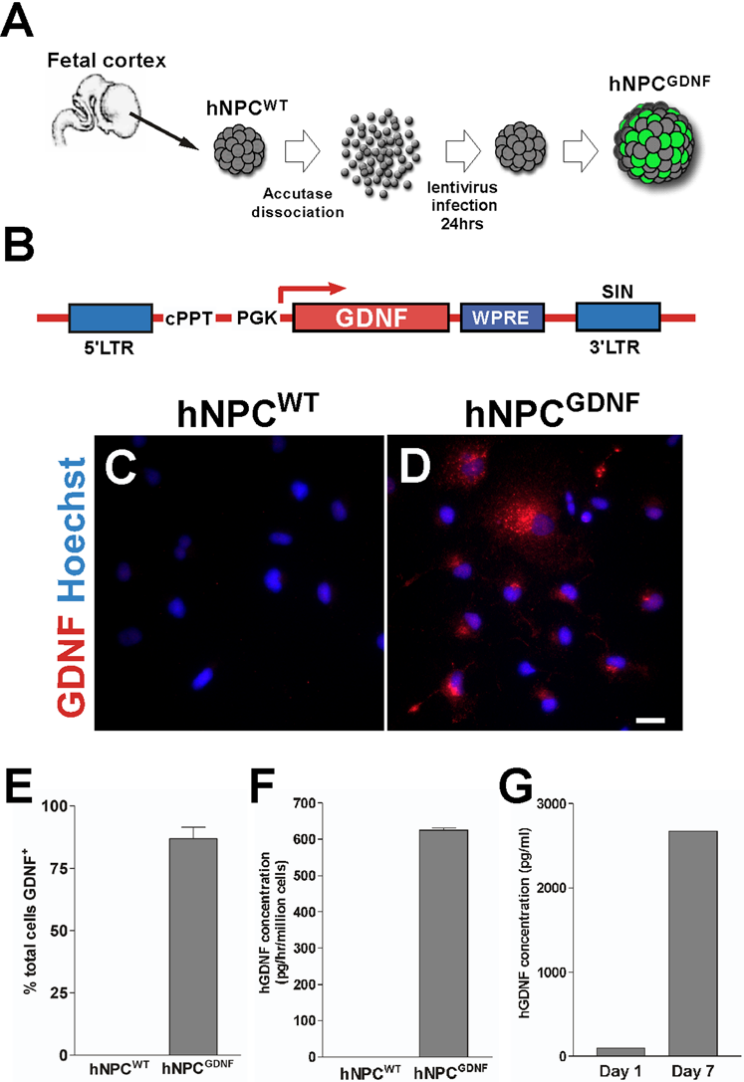
Modification of human neural progenitor cells to release GDNF by lentiviral infection. (A) Schematic illustration of GDNF expressing hNPC preparation. Naïve neural progenitor cells (hNPC^WT^) were infected with lentivirus to produce GDNF (hNPC^GDNF^). (B) Schematic illustration of lentivirus construct. LTR, long terminal repeat; PGK, mouse phosphoglycerate kinase 1 promotor; WPRE, post transcriptional regulatory element of woodchuck hepatitis virus; cPPT, central polypurine tract; SIN, self-inactivating. Photomicrographs of hNPC^WT^ (C) and hNPC^GDNF^ (D) show robust expression of GDNF protein (E). Approximately 87% of cells were shown to express GDNF. (F) hGDNF protein was detected by ELISA in the culture medium after lentivirus infection. (G) GDNF expressing hNPC did not down-regulate the transgene after differentiation, but rather accumulated it in the media over time. Scale bar: 20 µm.

We used a viral construct for constitutive expression of GDNF under the control of the mouse phosphoglycerate kinase 1 (PGK1) promoter (Figure 1). Posttranslational cis-acting regulatory element of the woodchuck hepatitis virus (WHV) is included and has been shown to significantly enhance transgene expression. The lenti-GDNF inactivated virus was produced as described previously [30]. High-titer stocks were obtained by ultracentrifugation, and the lentiviral particles were suspended in 1% bovine serum albumin in phosphate buffered saline. Particle content of viral batches was determined by p24 antigen ELISA (PerkinElmer Life Sciences, http://las.perkinelmer.com/). hNPC cultures were gently dissociated with Accutase (Innovative Cell Technologies, http://www.innovativecelltech.com/) to single cell suspension, washed extensively, counted and diluted to 1,000 cells/µl in culture medium. The cells were mixed with virus at a titer of 100 ng/p24/million cells and allowed to reform spheres in a 24 well plate. We could maintain this production for up to 20 weeks of expansion in culture [31]. For GDNF immunohistochemistry, some spheres were dissociated in a single cell suspension with Accutase for 10 min at 37°C. The resulting cells (25,000 cells/50 µl) were plated to glass coverslips coated with poly-L-lysine and laminin (Sigma-Aldrich) and cultured in the plating medium [maintenance medium with 2% B27 supplement (Invitrogen)] for 1 hour at 37°C before fixation. The plated cells were continuously maintained and differentiated for 7 days under serum-free conditions [26]. The concentrations of GDNF protein in the conditioned medium were measured by enzyme-immunoassays (human GDNF DuoSet, R&D systems).

### Cell transplantation

Long-term culture (10 weeks) of lenti-GDNF (hNPC^GDNF^) or wild-type (hNPC^WT^) human cortical neuropsheres were dissociated to single cells using Accutase and resuspended in the transplantation medium (49% Leibovitz L-15/49% PBS-glucose/2% B27 supplement) at a concentration of 120–180,000 cells/µl and maintained on ice. SOD1^G93A^ rats were anesthetized with isoflurane and the lumbar vertebrae were exposed and clamped in a spinal stereotaxic frame (David Kopf Instruments, http://www.kopfinstruments.com/) to maintain a steady position. Injection holes were drilled in laminae within L1 and L4 spinal cord segments, and the spinal cord was exposed. Cell suspension (120–180,000 cells in 1–2 µl, 4 sites per rat) was unilaterally injected 1.8 mm ventral from the dorsal dura surface at L1–4 using a 10 µl Hamilton syringe outfitted with a 75–100 µm tipped micropipette [28], [32]. Cyclosporine (*i.p.* 10 mg/kg, Novartis, http://www.novartis.com/) was administered daily beginning 3 days before surgery and until sacrifice.

### Immunohistochemistry

The animals were transcardially perfused with chilled 0.9% saline and 4% paraformaldehyde-phosphate buffered saline, and the spinal cords were collected. For neuromuscular junction analysis, tibialis anterior muscles of some animals were removed prior to fixation. Spinal cords were cryoprotected in 30% sucrose, and the lumbar regions were sectioned in 35 or 50 µm using a cryostat. One in six sections were processed for Nissl staining or immunostanined for human nuclei (hNUC, mouse monoclonal, 1∶200, Chemicon), human GDNF (goat polyclonal, 1∶250, R&D), and choline acetyl transferase (ChAT, goat polyclonal, 1∶200, Chemicon). After primary antibody incubation, biotinylated secondary (1∶200, Jackson Labs., http://www.jacksonimmuno.com/) and avidin–biotin method was followed by DAB (Vectors Laboratories Inc., http://www.vectorlabs.com/) development. Nickel ammonium sulfate enhancement was used for human GDNF immunostaining. For double labeling with hNUC, antibodies against human nestin (mouse monoclonal, 1∶200, Chemicon), glial fibrilly acidic protein (GFAP, rabbit polyclonal 1∶2000, Dako, http://www.dako.com/), ED1 (mouse monoclonal, 1∶500, Serotec, http://www.ab-direct.com/), Ki67 (mouse monoclonal, 1∶200, BD Biosciences, http://www.bdbiosciences.com/), or glutamate transporter GLT1 (rabbit polyclonal 1∶200, gift from Dr. Jeffrey D. Rothstein, Department of Neurology, Johns Hopkins University School of Medicine) was used. Primary antibodies were followed by secondary antibodies conjugated to Cy3 or Alexa Fluor 488 (anti-IgG, 1∶1000, Jackson Laboratories). For retrograde labeling of motor neurons, 2.5% True blue (Sigma-Aldrich) was injected into hind limb muscles 4 days prior to sacrifice. All images were optimized by using a fluorescence microscope or a laser scanning confocal microscope (Nikon, http://www.nikonusa.com/). To quantify astrocyte activation the number of GFAP positive cells on both sides of the spinal cord was counted using Metamorph Imaging software (Universal Imaging Corporation, http://www.moleculardevices.com), and presented as a percentage of the total number of counted cells, and compared in Prizm software (Graphpad software Inc., http://www.graphpad.com/). To compare microglial activation we measured the mean intensity of ED1 fluorescence in the ventral horn. Digital gray scale images were taken at fixed exposure time, magnification, and background/threshold parameters. The same size box was placed over the ventral horn region and integrated optical density of labeling expressed as gray pixel value was measured using SCION IMAGE software (Scion Co., http://www.scioncorp.com/). For the stereological analysis, the sections were prepared at 35 µm thickness, and every 6^th^ section was immunostained for hNUC. An unbiased estimate of the population of transplanted cells was obtained by using the optical fractionator probe within StereoInvestigator software (MBF bioscience, http://www.mbfbioscience.com/). This probe was used to estimate population size by systematically sampling known fractions within a region of interest. Equipment consisted of a Zeiss AxioPlan 2 microscope (Carl Zeiss, http://www.zeiss.com/) with a motorized x-y stage and a microcator for measuring movements in the z direction. Counts of hNUC positive cells were performed using a 100× oil-immersion objective. A grid size (400 µm×400 µm) was randomly placed on the tissue image which determined where the counting frames (15 µm×15 µm) were placed. Counting frames were superimposed on the image of the tissue and all cells stained with hNUC antibody, within the frame, were counted. Each section was traced and a 3D image was re-constructed by StereoInvestigator software. We also estimated Coefficient of Error using the software. Coefficient of Error represents a measure of the variance of the estimate from counting; which includes both the biological variation and the error introduced by the counting method. Coefficient of Error less that 0.1 indicates that the variance is sufficiently low enough to accept the estimation as accurate or at least not influenced by those two variables.

### Behavioral testing

Body weight measurements and all behavioral testing began at an age of 65 days and continued until endpoint. Rats were considered end-stage (endpoint) when they no longer exhibit reflexes allowing them to right themselves within 30 seconds. To analyze motor function in hSOD^G93A^ rats, the Basso-Beattie-Bresnahan (BBB) locomotor rating test and a beam walking test were performed twice a week. Open field locomotor scores were obtained in a small enclosure using the BBB locomotor rating scale as described previously [28], [29]. Briefly, each rat was allowed to walk around in a wading pool with a textured floor while we observed hind and fore limb movements for approximately 3 to 5 minutes. Each hind limb score was based on the 21 point scoring scale from no movement (0) to normal locomotion (21). Scoring takes into account paw rotation, toe clearance, weight support, the frequency of each, and the amount of movement occurring from each joint. The beam walking test was used to measure motor coordination and balance in ALS rats [29]. Briefly, rats were placed individually on one end of a seven-foot long multi-plane beam and allowed to run across. These different planes signified different scores based on hind limb placement while crossing the length of the beam. Each score was then recorded and summed for each hind limb to determine that limb's score. The total score was obtained by adding both hind limb scores. Cumulative survival statistics of disease onset was analyzed using the Kaplan-Meier method with Prizm software. Disease onset was estimated by using the BBB rating score of 17 or lower [29].

### Assessment of neuromuscular junction (NMJs) innervation

Tibalis anterior muscles were dissected and flash-frozen in supercooled isopentane. Muscles were sectioned at 20 µm using a cryostat and placed on glass slides for staining. The sections were fixed with 4% paraformaldehyde-phosphate buffered saline and labeled with alpha-bungarotoxin conjugated with fluorescence marker Alexa Fluor 594 (1∶1000, Invitrogen), anti-neurofilament 160 (mouse monoclonal, 1∶40, Sigma-Aldrich) and anti-synaptophysin (rabbit polyclonal, 1∶40, Dako) antibodies for 1 hour at room temperature. After washing with PBS, the sections were incubated with both anti-mouse and rabbit Alexa Fluor 488-conjugated secondary antibodies (1∶1,000, Jackson Laboratories) for 1 hour at room temperature. Following washing, the sections were covered with cover glasses using aqueous mounting medium (Immuotech, Marseilles, France). Axons and NMJs were imaged on a fluorescence microscope (Nikon) and Metamorph Imaging software (Universal Imaging Corporation). For quantitative analysis, we classified NMJs into three groups based on degree of innervation of postsynaptic receptor plaques by nerve terminals. Endplates were scored as “innervated” if there was overlap with the axon terminal, or “denervated” if the end-plate was not associated with an axon. Some neuromuscular junctions were associated with a preterminal axon only, or partial overlap between endplate and terminal. These were categorized as “intermediate”. About 50–60 endplates were exhaustively analyzed in each muscle from animals of each group. The number of endplates of each category was presented as a percentage of the total number of counted NMJs, and compared in Prizm software.

### Lumbar motor neuron count

Spinal cord sections (adjacent 5 sections to the injection sites) were mounted on slides and allowed to dry overnight. The slides were put through a gradual series of dehydration and re-hydration solutions, stained with Cresyl violet, differentiated, and coverslipped. Light microscopy images of the lumbar spinal cord were captured bilaterally with a digital camera (SPOT II, Diagnostics Instruments, http://www.diaginc.com/). A circular area-of interest was positioned over the ventral horn on both sides of the spinal cord. Area of interest, size, and position were standardized across sections by choosing a circular circumference that matched the curvature of the ventral horn. Each Nissl-stained neuron in the ventral horn with a distinct nucleolus and a darkly stained cytoplasm was manually traced using Metamorph Imaging software. Based on a pilot study, using motor neurons immunostained with ChAT or SMI32, α-motor neurons were defined as having cross-sectional area ≥700 µm^2^. All α-motor neurons were counted in the selected area of the ventral horn.

### GDNF ELISA

hNPC^GDNF^ and hNPC^WT^ were bilaterally (120,000 cells in 1 µl, 2 sites per one side) injected in the lumbar spinal cord of a wild-type female rat as described above. Four regions (1 cm each) from both the transplanted and outside the transplant were dissected and flash-frozen in supercooled isopentane. The tissues were then suspended in 1 ml of lysis buffer [26], incubated for 15 min on ice, centrifuged for 15 min at 10,000 *g* at 4°C and stored at −80°C. Protein concentration was determined by a detergent-compatible modified Bradford assay (Bio-Rad, http://www.bio-rad.com/). The concentrations of GDNF protein were measured by ELISA as described above.

## Results

### hNPC can be modified to release GDNF and ameliorate early motor neuron loss in hSOD^G93A^ rats

hNPC were isolated from the cortex of 15 week old human fetal tissue under the guidelines set out by NIH for collection of such tissues, and local IRB approval. Following expansion as spherical neurospheres [24], they were infected with a lentiviral construct encoding GDNF under the control of phosphoglycerol kinase (PGK) promoter (Figure 1B). Approximately 87% of hNPC^GDNF^ cells expressed the GDNF protein based on immunocytochemistry (Figure 1C–E) which was released into the media at a rate of 618 pg/hr/million cells (Figure 1F). Non-infected hNPC (wild-type hNPC, hNPC^WT^) did not release detectable amounts of GDNF. Release of GDNF from the cells was maintained following 1 and 7 days differentiation into neurons and astrocytes (Figure 1G).

**Figure 2:**
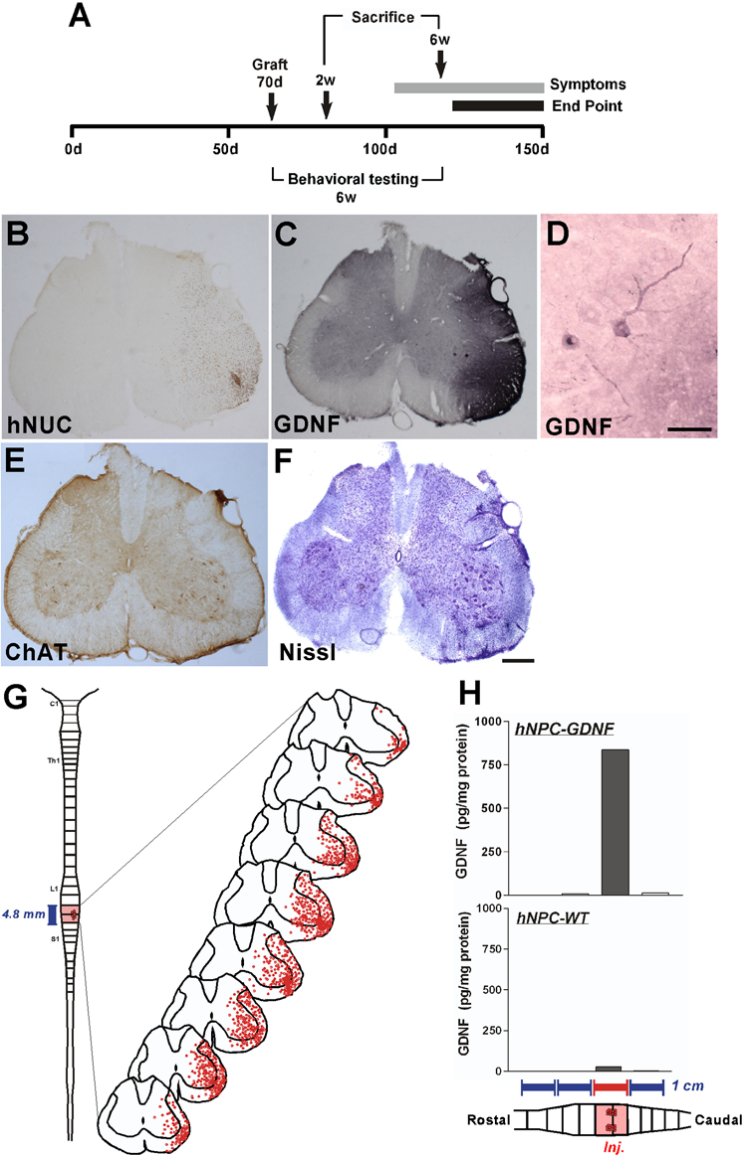
Survival and GDNF expression of transplanted hNPC^GDNF^ in the spinal cord of the SOD1^G93A^ rats. (A) Rats were transplanted with hNPC at day 70 and sacrificed at either 2 or 6 weeks post transplantation. (B) Staining with the human specific marker (hNUC) showed unilateral survival of cells in the ventral grey and white matter. (C) Surviving cells release GDNF. (D) Some endogenous rat motor neurons were decorated with GDNF. (E) ChAT and (F) Nissl staining showed increased motor neuron number around the transplant site. (G) There was widespread distribution of hNPC in the lumbar region of the spinal cords 6 weeks after transplantation. (H) GDNF expression was detected only in the area of the cell transplant. Scale bars: 1 mm in F; 100 µm in D.

We next wanted to assess whether hNPC^GDNF^ could affect motor neuron survival following transplantation into the spinal cord of SOD1^G93A^ rats. We have previously shown that SOD1^G93A^ founder rat colonies show disease onset at approximately either 105 days (early onset) or 160 days (late onset) in addition to a gender specific effect with males showing an earlier onset than females [29]. Although we did not have any way to predict which onset type they would have prior to transplantation, we only used female animals to reduce sex based variation. hNPC^GDNF^ (182,000 cells/µL×2 µL×4 unilateral sites in L1/L2) were transplanted into the lumbar spinal cord of SOD1^G93A^ rats (n = 6 females for each group) at 70 days of age (Figure 2A). Although a full behavioral assessment was not performed on this group of animals, none showed any overt signs of limb paralysis at 6 weeks after grafting (112 days) suggesting that this would have been a late onset cohort. Animals were sacrificed at either 2 or 6 weeks post transplantation. Three rats in the 2 week survival group and 4 rats in the 6 week survival group were found to contain human cells based on the specific human nuclear antigen (hNUC). Surviving cells were well targeted and had migrated out unilaterally into degenerating regions of the ventral horn in all of these animals (Figure 2B). The transplants spread anteriorally and posteriorally up to 7.2 mm (4.8±3.9 mm at 2 weeks and 4.4±0.9 mm at 6 weeks post transplantation; mean±SEM) from the injection sites (Figure 2G). There were no adverse effects in any of the animals and no sign of excessive cell growth indicative of tumor formation. In one of the hNPC^GDNF^ treated animals we also performed a stereological analysis to count the total number of hNUC positive cells at 6 weeks post transplantation. Approximately 1,456,000 cells were transplanted and 1,629,750 cells were estimated to be within the spinal cord using stereological counting methods. As clearly shown in Figure 2G, the transplanted cells migrated out within one side of the lumbar spinal cord in this rat and did not cross the midline.

We next checked GDNF release from these cells using immunocytochemistry and found that the location of hNUC positive cells correlated exactly with the release of GDNF into the surrounding area (Figure 2C). In regions of GDNF delivery many host motor neurons were labeled with GDNF suggesting active uptake of the protein into surviving motor neurons (Figure 2D). We also determined GDNF released from the transplanted cells in the spinal cord using ELISA. At 6 weeks following transplantation of 3 more animals (480,000 cells total), 1 cm regions from both inside and outside the transplant were dissected, homogenized, and then processed for GDNF ELISA. Significant amounts of GDNF (up to 820 pg/mg tissue protein) were detected in the region of the spinal cord with the transplants, but not outside (Figure 2H). Animals transplanted with wild type hNPC did not show any detectable GDNF release.

**Figure 3:**
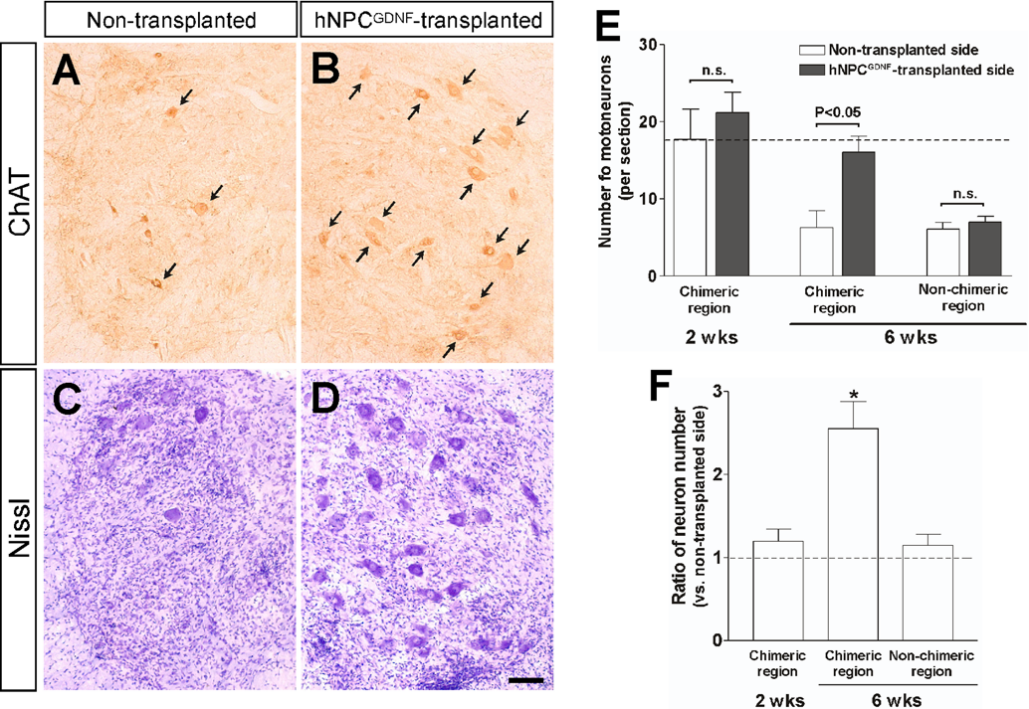
Motor neurons are protected by GDNF secreting hNPC transplants in the spinal cord of SOD1^G93A^ rats. The number of motor neurons was higher in hNPC-transplanted (chimeric) side (B, D) compared to the contralateral non-grafted (non-chimeric) hemisphere (A, C). Cell number reduced by 70% at 6 weeks post grafting in sham treated SOD1^G93A^ rats (E). There was almost complete protection of motor neuron loss within chimeric (transplant) regions of the spinal cord. Outside of these transplant (non-chimeric) regions motor neuron numbers dropped to less than 70%. The broken line indicates the number of motor neurons in wild-type rats. (F) The ratio of motor neuron numbers compared to the contralateral side. *: P<0.05 vs. 2 wks and 6 wks outside groups. Scale bar: 100 µm in D.

In order to establish whether GDNF secreting hNPC could ameliorate motor neuron loss, we counted the number of Nissl stained or choline acetyltransferase (ChAT) positive cells both within the region of the transplant and outside the region of the transplant. There was very little loss at 2 weeks post grafting (94 day old animals) in either the transplanted or non transplanted side when compared to wild type animals (Figure 3E) suggesting that motor neuron degeneration had not yet reached significant levels in this late onset cohort of animals. However, by 6 weeks post grafting (112 day old animals) there was a significant 70% reduction in motor neuron cell number in the non-transplanted side of the SOD1^G93A^ rat which was almost completely prevented within chimeric regions of the spinal cord (Figure 3E, F). Outside of these chimeric spinal cord regions there was no protection of motor neurons (Figure 3E, F). Thus, even within the same animal, hNPC^GDNF^ can provide local protection of motor neurons from cell death.

### Migrating hNPC^GDNF^ express nestin but not GFAP or GLT1 and do not affect host reactive astrocytes

**Figure 4:**
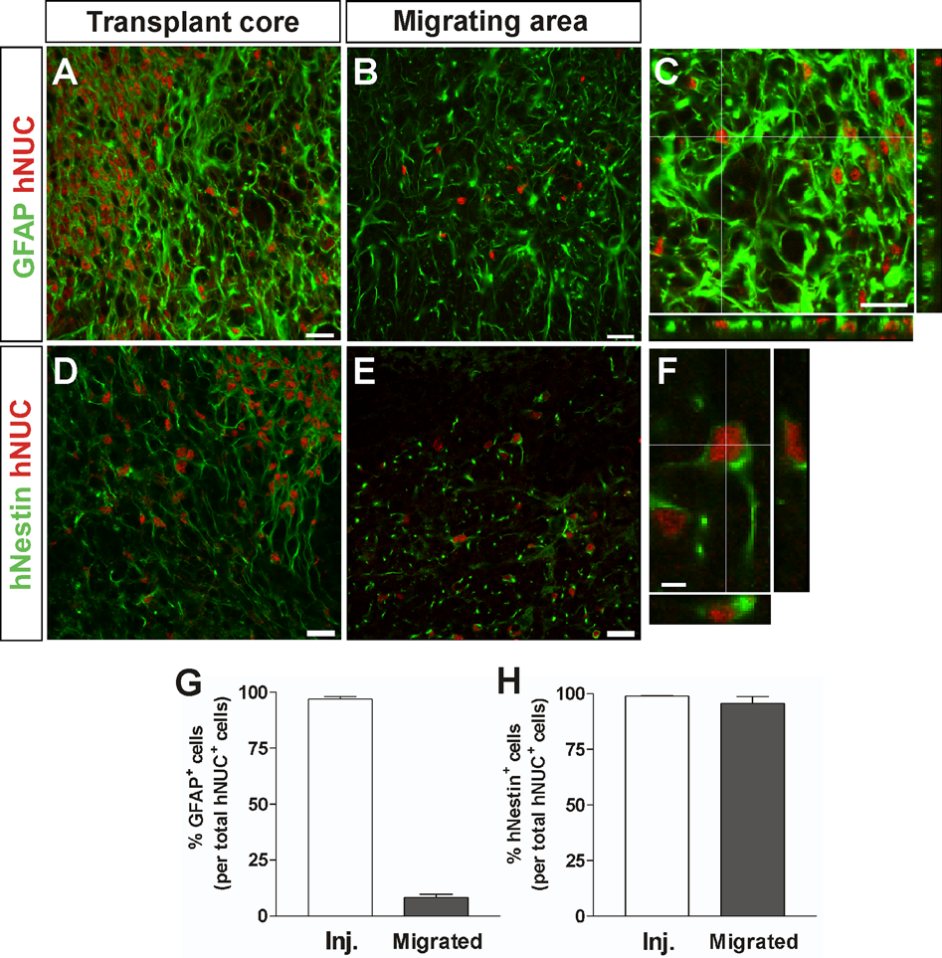
Migrating hNPC^GDNF^ express nestin but not GFAP. Confocal stacked images through sections show hNPC were GFAP positive within the core of the transplant (A, C, G). However, away from the site of the transplant GFAP expression was lost (B, G). Continual nestin expression was observed in both the transplant core and the region of migrating cells (D, E, F, H). Scale bars: 20 µm in A–E; 5 µm in F.

It was possible that the hNPC^GDNF^ were generating glial cells that provided additional protection to the dying motor neurons [19]. In the 6 week transplant group, human cells within the transplant core were found to remain GFAP^+^ (Figure 4A, G) and Nestin^+^ (Figure 4D, H). However, following migration, the majority of cells (>95%) retained nestin expression (Fig.4E, H) but down regulated GFAP (<10%) (Figure 4B, G). Other labeling showed that these migrating cells did not express two other astrocyte markers vimentin or S100, suggesting that they were not reactive astrocytes. They were also negative for neuronal markers such as TuJ1 (data not shown). Glutamate transport by glial cells may protect motor neurons in rodent models of familial ALS [33]. However, the hNPC^GDNF^ did not express GLT1 and furthermore we found that host cells surrounding the dying motor neurons expressed high levels of GLT1 in non transplanted and transplanted regions of the spinal cord (Figure S1). This is in contrast to other studies that have shown down regulation of this transporter in the same model [5]. Together, this data suggests that the hNPC that migrated along side the dying motor neurons were not reactive GFAP expressing astrocytes with glutamate transporters, but rather immature nestin positive progenitors.

**Figure 5:**
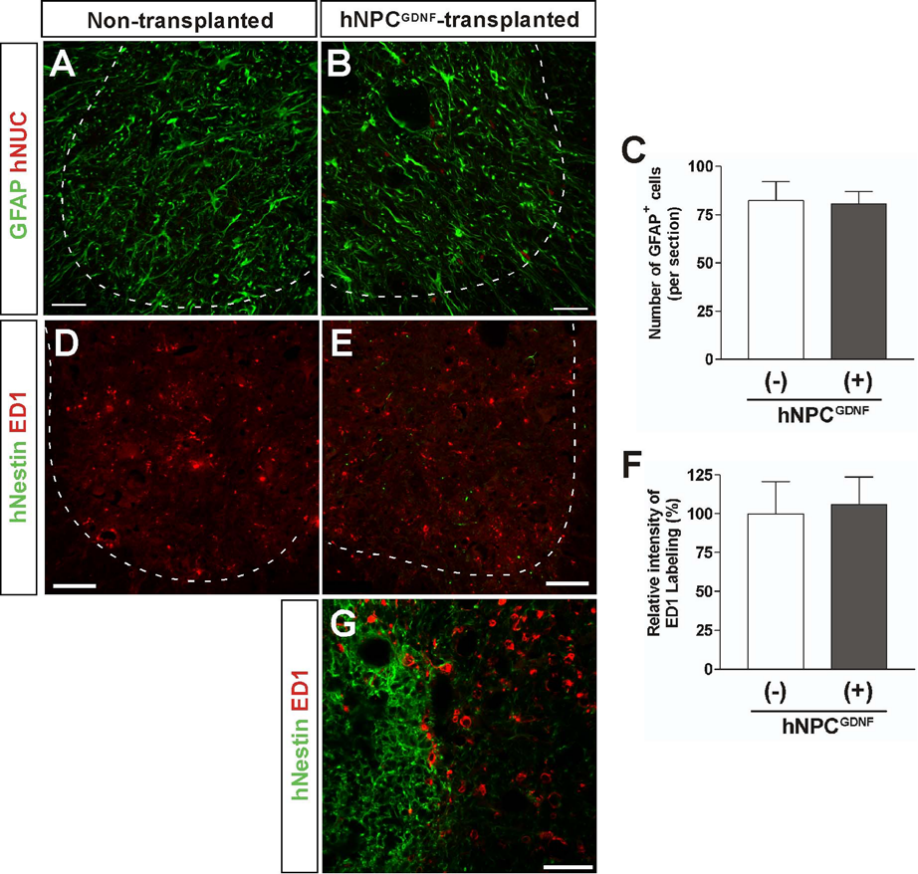
Glial cell activation is not changed following hNPC^GDNF^ transplantation in the spinal cord of SOD1^G93A^ rats. Photomicrographs of the non-transplanted (A) and the hNPC-transplanted (B) indicate no change in number of GFAP positive reactive astrocytes in the ventral root of the spinal cord (C). Similarly, there was no difference in the level of microglia activation within regions of motor neuron protection (D–F). However, a detailed analysis using higher magnification (G) revealed microglia activation in the transplant core (the left side of a broken line indicating the border between the transplanted core). Scale bars: 50 µm in A, B, D, E; 20 µm in G.

Activation of host glial cells is one hallmark of ALS, and increases with disease progression in the SOD1^G93A^ rat which may be either protective or contribute to motor neuron death [16]. The number of host GFAP reactive astrocytes (not hNUC labeled) in the grafted vs. non-grafted side at 6 weeks post transplantation was the same (Figure 5C). This suggested that the protective effects of hNPC^GDNF^ were not mediated through a modulation of host reactive astrogliosis. We also determined the levels of microglia activation by using immunostaining for reactive microglial marker ED1. There was no difference in the level of microglia activation within regions of motor neuron protection away from the core of the transplant (Figure 5E, F). However, there were reactive microglia in the transplant core (Figure 5G) which may suggest a low grade rejection phenomenon at high cell density as we have reported previously in cyclosporine treated animals [34]. This may also explain why some rats had no surviving transplants.

### hNPC^GDNF^ transplants do not lead to a reduction in ipsilateral limb paralysis in SOD1^G93A^ rats showing early onset

**Figure 6:**
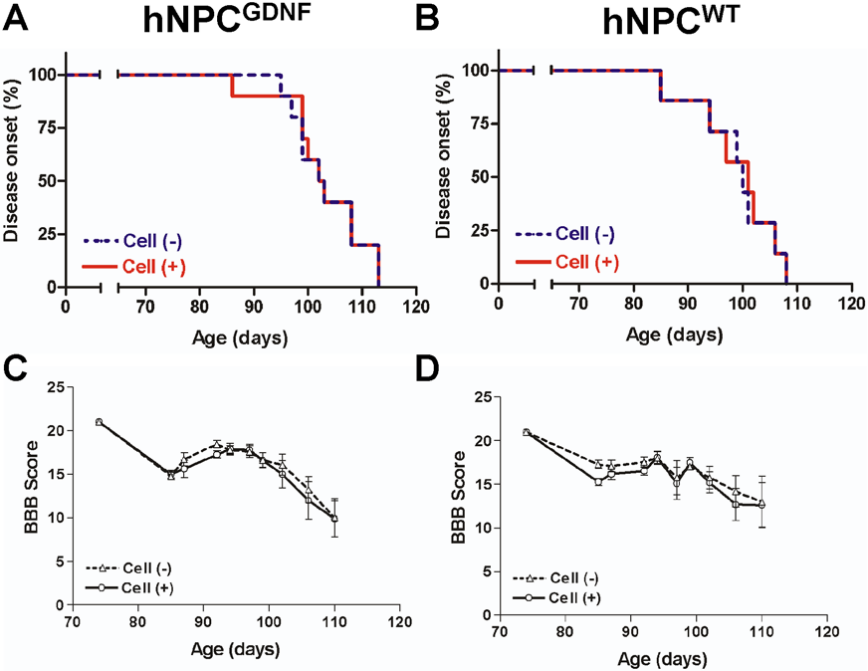
Disease onset and ipsilateral limb function was not affected by hNPC transplants in SOD1^G93A^ rats. Kaplan-Meier curves of disease onset in the animals with hNPC^GDNF^ (A) or hNPC^WT^ (B) transplants. Basso-Beattie-Bresnahan (BBB) locomotor rating scores (C, D) showed that a significant difference was not observed in between the transplanted side and the non transplanted side in any group.

We next wanted to assess whether the hNPC^GDNF^ transplants resulted in any positive or negative effects on limb function in the SOD1^G93A^ rat, and dissociate the effects of GDNF release from the effects of the cells themselves. For these studies a second cohort of female SOD1^G93A^ rats were unilaterally transplanted at 70 days with either hNPC^WT^ (n = 12), hNPC^GDNF^ (n = 12) or just received vehicle saline infusions (control; n = 6). In contrast to the first cohort of animals, distinct hind limb changes were noticed at early time points suggesting an early disease onset in this group. However, there was no significant difference in hind limb disease onset between the transplanted side and the non-transplanted side in any animal (Figure 6A, B). We also tested individual limb function using the Basso-Beattie-Bresnahan (BBB) rating test where both groups of animals showed similar disease progression up to 115 days of age (Figure 6C, D). Finally, we used a beam walking test to measure motor coordination and balance after onset and again found no effect of the transplant on this behavioral task (data not shown). Together, this data shows that while there was no detrimental effect of the transplant on motor function, there was no apparent benefit either. Furthermore, it was also clear that this cohort showed an aggressive and early onset of disease and rapid progression when compared to the cohort used for anatomical studies above, such that 5 animals from the hNPC^WT^ group and 4 from the hNPC^GDNF^ group were at disease end stage before the 6-week post graft date had been reached.

### GDNF released by hNPC can maintain healthy cholinergic motor neurons within chimeric spinal regions until disease end point, but have no effect on loss of muscle innervation in SOD1^G93A^ rats

**Figure 7:**
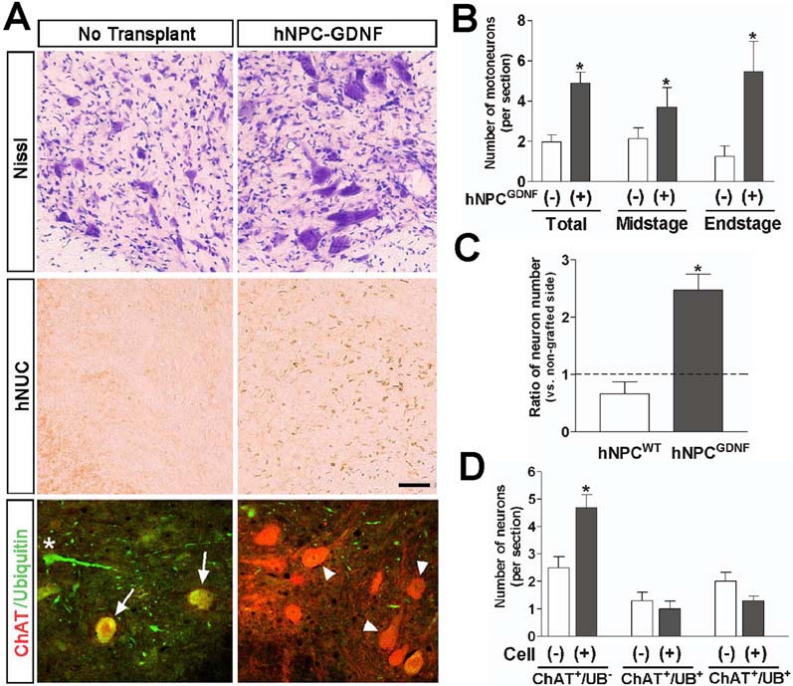
GDNF released from the hNPC is critical for motor neuron survival in this model. (A) Even though a number of animals in the hNPC^GDNF^ group reached end stage, the number of motor neurons was higher in hNPC-transplanted side compared to the contralateral non-grafted hemisphere. Surviving hNPC were found in the adjacent section of the transplanted side, but not in the contralateral side. We also double labeled sections throughout chimeric regions of each animal for both ChAT (red) and ubiquitin (UB; green), While the non transplanted side contained many ChAT^+^/ubiquitin^+^ (early degenerating motor neuron; indicated by arrows) and ChAT^−^/ubiquitin^+^ (late degenerating motor neuron; a star), most of the surviving ChAT^+^ motor neurons within chimieric regions of the transplanted spinal cord did not express ubiqutin (designated by arrow heads). (B) Motor neuron counts showed that there was a significant increase in motor neuron survival at both early and late time points within chimeric spinal cord regions rich in GDNF (n = 9 for total, 4 for mid stage and 5 for end stage). (C) While wild type hNPC not releasing GDNF did not affect motor neuron cell death in this model, there was approximately 2.5 fold increase compared to the non-grafted side in motor neuron survival in the hNPC^GDNF^ group. *: P<0.05 vs. hNPC^WT^. (D) Cell counting using sections double labeled for both ChAT and ubiquitin revealed that secreted GDNF from the hNPC was able to maintain healthy cholinergic motor neurons up until disease end point in this model of familial ALS. *: P<0.05 vs. cell (-). Scale bar: 50 µm.

The animals used for behavioral testing above were sacrificed at either 6 weeks or disease end point, whichever came first. Six out of the 12 animals with hNPC^WT^ had surviving transplants while 9 out of 12 animals with hNPC^GDNF^ had surviving transplants. The average number of surviving motor neurons in the non-transplanted side was approximately 2.5±0.7 per section (compared to 6.3±2.2 in the first cohort of animals) further suggesting a more aggressive disease progression in this group. However, there was a highly significant 2.5 fold increase in motor neuron survival within the hNPC^GDNF^ group when compared with the non grafted side (P<0.01; Figure 7B, C). A number of animals (n = 4) in the hNPC^GDNF^ group reached disease end stage during the 6 week period and were analyzed separately. Even at this time point the number of motor neurons remained significantly higher within chimeric regions of the spinal cord when compared to the contralateral side (5.5±1.5 in the transplanted and 1.3±0.5 in the non-transplanted side; Figure 7B, C; P<0.05). Interestingly, there was no significant difference in the number of motor neurons within chimeric regions of the hNPC^WT^ group and the non-transplanted side (2.1±0.7 in the transplant side and 3.1±1.3 in the non-transplant side, Figure 7C) showing that GNDF release from the cells is responsible for the increased motor neuron survival.

Next we double labeled sections throughout chimeric regions of each animal for both ChAT and ubiquitin, one marker of degenerating motor neurons in this model [19]. Most of the large surviving ChAT^+^ motor neurons within chimeric regions of the transplanted spinal cord did not express ubiqutin (P<0.01; Figure 7A, D). Both the transplanted and non transplanted side also contained a number of ChAT^+^/ubiquitin^+^ (early degenerating motor neuron) and ChAT^−^/ubiquitin^+^ (late degenerating motor neuron). Although there was a trend for more of these in the non transplanted side the differences were not significant (P<0.07). Clearly there is death of cholinergic neurons even in the presence of GDNF, but those motor neurons that are protected are not undergoing degeneration based on ubiquitin staining. Together, these data show that secreted GDNF from hNPC was able to maintain cholinergic motor neurons up until disease end point in this model of familial ALS. In fact, the degree of survival was similar to the number of motor neurons seen at earlier pre symptomatic stages shown in the first cohort of animals (6.3±2.2). Finally, we also assessed whether GDNF may have reduced the expression of human mutant SOD1 protein in surviving motor neurons. It was clear that all remaining cholinergic motor neurons protected by GDNF continued to express human mutant SOD1 (data not shown).

**Figure 8:**
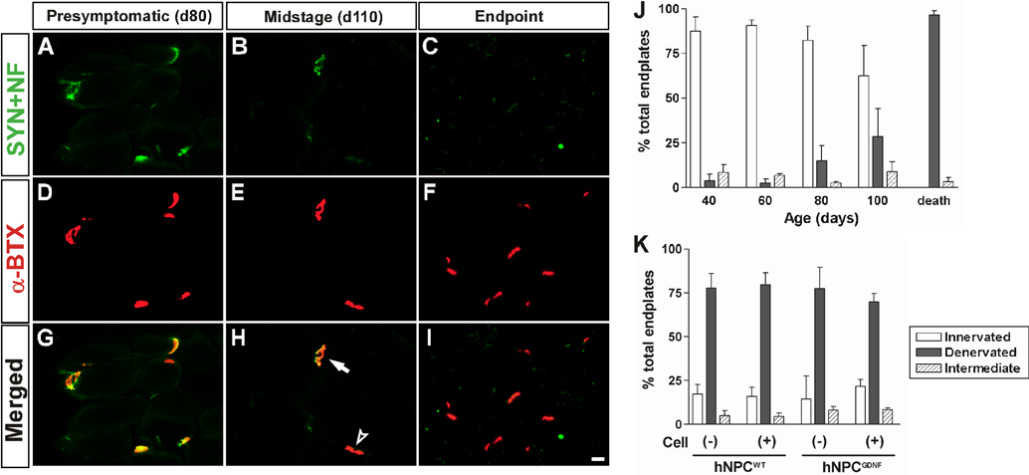
Neuromuscular junction innervation following hNPC transplantation. Axons were identified with synaptophysin and neurofilament (SYN+NF), motor endplates were identified with α-bungarotoxin (α-BTX). (A, D, G) In pre-symptomatic animals (80 days old), most endplates were innervated (yellow). (B, E, H) The number of denervated (an arrow head) endplates increased at the mid-stage (110 days old), although a few were still innervated (a white arrow). (C, F, I) All endplates were denervated at end-stage. (J) In the pre-symptomatic animals (40, 60, and 80 days old), over 80% of endplates were innervated. The number of denervated endplates was gradually increased by the mid-stage (100 days old), and most of endplates were denervated at end-stage. (K) Detailed endplate counts showed that there was no significant effect of hNPC^GDNF^ on the innervation of neuromuscular junctions in the hindlimb muscle of animals showing increased numbers of motor neurons in the spinal cord. Scale bar: 20 µm.

Given the extent of protection by GDNF, we were surprised that this did not result in recovery of some limb function. Previous studies using the *pmn* mouse model [35] and the Bax-deficient mice crossed with mice expressing mutant SOD1^G93A^ [36] have shown that GDNF can protect motor neurons which are no longer connected to the muscle. We therefore analyzed neuromuscular junctions (NMJs) in the animals that had shown increased motor neuron survival within the spinal cord (Figure 8). Using a combination of hNPC transplants and intramuscular injection of retrograde tracer True Blue, we first confirmed that the region of muscle analyzed received projections from the chimeric spinal cord region (see Figure S2). The level of innervation in hind limb muscles (tibialis anterior) was estimated by using double staining for axons and motor endplates. We classified NMJs into three groups (innervated, denervated, and intermediate) based on degree of innervation of post synaptic receptor plaques by nerve terminals. In the pre-symptomatic animals up to 80 days old, over 80% of endplates were innervated (Figure 8A, D, G, J). The number of denervated endplates was gradually increased by the mid-stage (Figure 8B, E, H, J), and all endplates were denervated at end-stage (Figure 8C, F, I, J). Interestingly, we found that there was no significant effect of hNPC^GDNF^ on the innervation of neuromuscular junctions at the hind limb muscle in the 4 animals showing increased numbers of healthy cholinergic motor neurons in the spinal cord (Figure 8K). We conclude that while hNPC releasing GDNF were able to protect motor neurons, they were no longer connected to the muscle and thus not improving ipsilateral limb function in this rat model of FALS.

## Discussion

Our data show that transplantation of hNPC releasing GDNF can have dramatic effects on motor neuron survival in the SOD1^G93A^ rat model of familial ALS. At early, pre symptomatic stages of the disease, GDNF was able to maintain nearly 100% of the motor neurons which otherwise would have reduced to only 30% of normal values (Figure 3). Even at symptomatic disease end stage GDNF was able to maintain over 30% of the motor neurons, a level which should be sufficient to prevent symptoms. However, muscle innervation was not maintained in these same animals and no changes in limb function were observed.

### GDNF release from hNPC increases motor neuron survival

Direct gene therapy to the spinal cord using the same lenti-GDNF construct described in the current study has previously been attempted with no protective effect on lumbar motor neurons in the mutant SOD1 mouse, even though high levels of GDNF secretion was achieved [14]. Direct gene therapy involves the infection of motor neurons that are undergoing degeneration and it is possible that this approach induces a high level of stress that reduces any possible neuroprotective effects [37]. Since in the current *ex vivo* gene therapy approach used hNPC to deliver the protein, host motor neurons did not need to be exposed to lentivirus which may have increased the chance of neuroprotection. Furthermore, other factors secreted by the hNPC may have worked with GDNF to improve survival.

There are a number of ways in which GDNF produced by hNPC may modulate motor neuron survival. In ALS, glial activation has been described in the spinal cord of patients [38] and SOD1 mice [4]. Assuming that reactive gliosis occurs in response to motor neuron death, it was possible that rescue of a substantial fraction of neurons would have reduced the extent of the reactive gliosis on the grafted side. However, we found that astrogliosis was not reduced and we observed highly reactive microglia throughout the spinal cord in both sham treated and transplanted animals at end stage. Thus, the protective effects of GDNF were not mediated through a modulation of host reactive astrogliosis, and GDNF induced the survival of motor neurons expressing mutant SOD1 protein within a highly reactive environment. We were surprised to find that most cholinergic motor neurons protected by GDNF had not even started to express early markers of cell death such as ubiquitin. We suggest that GDNF, perhaps in combination with other factors secreted by hNPC, activates cell protection pathways within host motor neurons that may be linked to anti-apoptotic proteins [39], [40].

### hNPC alone have no effect on motor neuron survival

Our studies were prompted by elegant data from chimeric mice showing that mutant SOD1-overexpressing neurons surrounded by normal glia remained relatively intact, whereas normal neurons surrounded by mutant SOD1-overexpressing glia showed signs of damage [17]–[19]. We initially hypothesized that transplanted hNPC not releasing GDNF would give rise to fresh glial cells, and that the chimeric regions would show decreased motor neuron death. However, wild type hNPC had no effect on motor neuron survival (Figure 7A). This could be due to a number of factors. First, we did not see any GFAP expression in cells that had migrated into regions of motor neuron degeneration which instead retained the progenitor cell marker nestin. This suggests lack of maturation or slow development of the human cells which we have seen previously following transplantation of hNPC into the striatum [24]. There is also a wide heterogeneity of astrocyte types which is affected by strain [41] and region from which the progenitor cells were isolated [42]. As mentioned below, other groups have shown hNPC derived from the spinal cord can produce GDNF [43] and in our own studies hNPC derived from the mesencephalon also produce GDNF (Kim and Svendsen, unpublished observations). Thus, perhaps astrocytes from cortically derived hNPC are not able to support spinal cord motor neurons. Astrocytes are essential partners of motor neurons, not only providing them with trophic support, but also mediating rapid recovery of synaptic glutamate through the action of the glial glutamate transporter GLT1 [44]. Interestingly, hSOD1^G93A^ mice heterozygous for GLT1 develop earlier-onset disease, while drugs that increase GLT1 activity extend survival [45]. While we did see expression of EAAT2 (human GLT1 homologue) in hNPC *in vitro*, both the core and surrounding regions of the transplant were totally negative for this transporter based on immunocytochemistry (Figure S1) suggesting down regulation *in vivo*. However, we were surprised to find that even at end stage there was expression of the GLT1 transporter using immunocytochemistry in the ventral horn surrounding the dying motor neurons, both inside and outside of the transplant region. This is in direct contrast to a previous report in the same rat model [5] although other groups have also failed to see reduced GLT1 expression in mouse models of ALS [46]. While there may be technical differences with regard to antibody sensitivity, we would confirm this later study, and suggest that in this model expression of at least low levels of GLT1 is not sufficient to prevent motor neuron death. It would however be very interesting to express higher levels of GLT1 and see if this has protective effects on these motor neurons. Together, our data show that while hNPC can survive and integrate they do not produce mature astrocytes that can prevent the death of motor neurons.

Other recent studies have shown more positive effects on limb function when human neural progenitor cell cultures derived from the developing spinal cord have been transplanted into the SOD1^G93A^ rat [43]. While the cells appeared to extend the onset and endpoint of disease to a small degree, there was no control for possible gender influences (male vs. female ratio was not given) or drift in the colonies that are prominent with this animal model [29], [47]. This study also suggested an increased survival of motor neurons in chimeric regions in a small subset of three animals per group, even in the absence of maturation into GFAP positive astrocytes [43]. Regional specificity of neural stem cells has been shown previously [48]–[50] and therefore it is possible that spinal cord derived neural stem cells have different properties following transplantation. Interestingly, spinal cord neural stem cells spontaneously release small amounts of GDNF and produce neurons after grafting [43] in contrast to more extensively passaged cortical derived neural progenitor cells used in the current study which do not produce GDNF or neurons after grafting [27], [28]. Thus, GDNF may have been responsible for the effects on motor neuron survival in this study. While a direct comparison between cortical and spinal cord derived neural progenitor cells within the same experiment would be interesting, from a practical point of view human neural progenitor cells derived from the spinal cord are more difficult to expand than those from the cortex [51] and may therefore be less useful for future clinical studies. More modest effects on function with little detailed analysis of motor neuron survival have been seen when mouse sertoli cells [52] or hNT neurons [53] derived from a human teratocarcinoma line were transplanted into mouse models of FALS. In both cases the cells produced GDNF which again may have contributed to the effects.

### Healthy surviving motor neurons do not maintain contact with muscle fibers

Connection to the muscle at the neuromuscular junction is lost in ALS mouse models long before motor neuron degeneration or death and the initiation of symptoms, leading to the hypothesis that ALS may be a “dying back” axonopathy [54], [55]. A similar phenomenon has been reported in previous experiments where GDNF could protect motor neurons that had retracted from the muscle in the *pmn* mouse model [35]. Furthermore, deletion of the proapoptotic protein, Bax, has been shown to completely protect motor neurons in the ALS mouse model that have no distal connections to the muscle [51]. To address this question we analyzed motor endplates which showed a similar, but less rapid decline in number to that found in the mouse model. We found that motor neurons surviving in the spinal cord due to GDNF delivered by the hNPC did not reduce the number of denervated muscle end plates. Our results suggest that protection of both cell bodies and nerve terminals in the muscle may be required for functional limb improvements in this model of familial ALS, and provides further data in the rat supporting previous mouse studies showing that the survival of the motor neuron can be independent of muscle innervation [51]. An interesting recent study showed that in a viral model of motor neuron degeneration, axonal outgrowth and attachment to the muscle could be achieved from transplanted motor neurons by using GDNF secreting cells implanted into the sciatic nerve [56] and that insulin-like growth factor 1 (IGF1) may have specific effects on motor neuron fiber outgrowth [57]. We feel that these types of combinatorial approaches of cells and different growth factors may improve muscle connection and lead to potential improvements in limb function.

### Potential for ameliorating motor neuron loss in ALS

Before considering using stem cells for transplantation into patients it is essential to show that there is minimal risk of tumor formation, survival within a diseased environment and low levels of adverse functional effects. Neural tissues derived from mouse ES cells can form teratomas in the brain [58]. Furthermore, a recent report showed that the grafts derived from human ES cells exhibited expansion cores of undifferentiated mitotic neuroepithelial cells [59]. Fetal-derived hNPC described in the current studies divide for only a limited time following transplantation into the striatum [34] and have not been shown to form tumors in a large number of animal studies. In the current work we confirm that there was no sign of mitotic cells or tumor formation within the grafted area of SOD^G93A^ rats. Furthermore, there were no adverse effects on function, even when high numbers of cells were found to survive. It is remarkable that given the severely degenerative xenograft environment, the human progenitor cells were still able to survive, integrate and release GDNF that had a protective effect on motor neurons in this model of familial ALS. Given the major issues for delivering this and other growth factors to specific regions of the spinal cord and brain we feel this is an important new approach to drug delivery in ALS patients that may also be applicable in other disorders [60]. While we conclude that hNPC secreting GDNF may represent a viable source of cells for protecting dying motor neurons, further animal studies are clearly needed to slow the loss of muscle innervation and thus increase the chances of functional recovery following transplantation.

## Supporting Information

Figure S1 Immunostaning of reactive microglia marker ED1 and glutamate transporter GLT1. The grafted hNPC do not express GLT1 in both the surrounding (A) and core (B) regions of the hNPC transplant. (C) A detailed analysis using higher magnification revealed microglia activation in the transplant core (the left side of a broken line indicating the border between the transplanted core). Scale bars: 100 µm in A; 50 µm in B; 20 µm in C.
(1.94 MB TIF)

Figure S2 The correlation with the tracer labeled motor neurons and hNUC staining. hNPC were unilaterally transplanted into the lumbar spinal cord of wild type rats. After surgery, a retrograde tracer True Blue (2.5%) was injected into the same side as the hNPC transplants of the tibialis anterior muscle. Four days after transplantation, the spinal cord was collected and checked for true blue positive neurons in the lumbar spinal cord (A). Furthermore, the adjacent section was immunostained with hNUC antibody (B). Scale bars: 100 µm.
(1.74 MB JPG)
