# Supplementary figures and images for: GDNF Secreting Human Neural Progenitor Cells Protect Dying Motor Neurons, but Not Their Projection to Muscle, in a Rat Model of Familial ALS
Source: PLoS One. 2007 Aug 1;2(8):e689. doi: 10.1371/journal.pone.0000689 (PMC1925150; doi:10.1371/journal.pone.0000689)

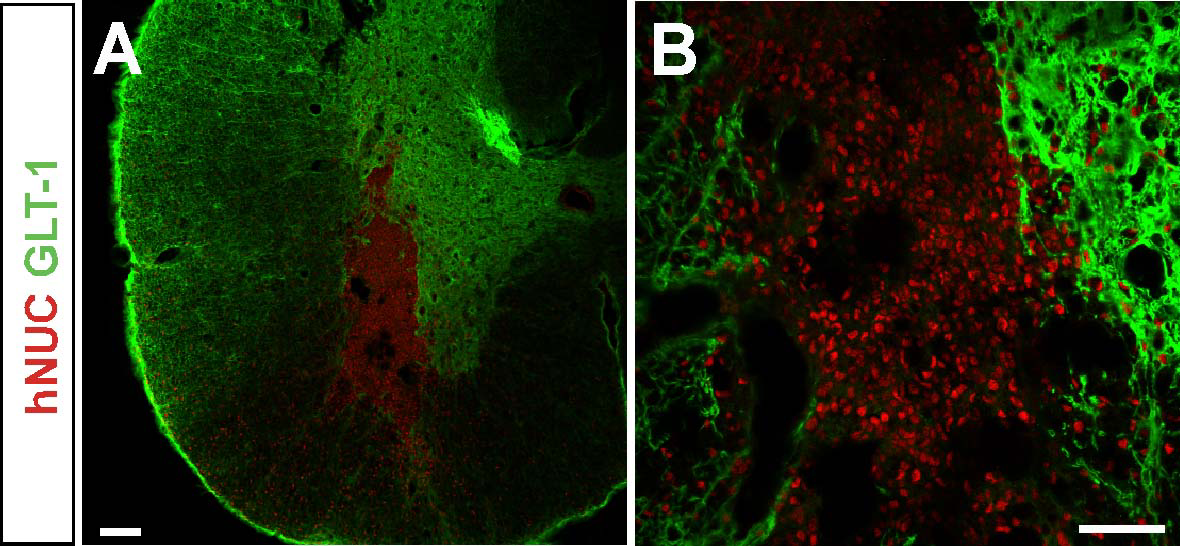

Supplement: Figure S1 — Immunostaning of reactive microglia marker ED1 and glutamate transporter GLT1. The grafted hNPC do not express GLT1 in both the surrounding (A) and core (B) regions of the hNPC transplant. (C) A detailed analysis using higher magnification revealed microglia activation in the transplant core (the left side of a broken line indicating the border between the transplanted core). Scale bars: 100 µm in A; 50 µm in B; 20 µm in C. (1.94 MB TIF) [file pone.0000689.s001.tif]

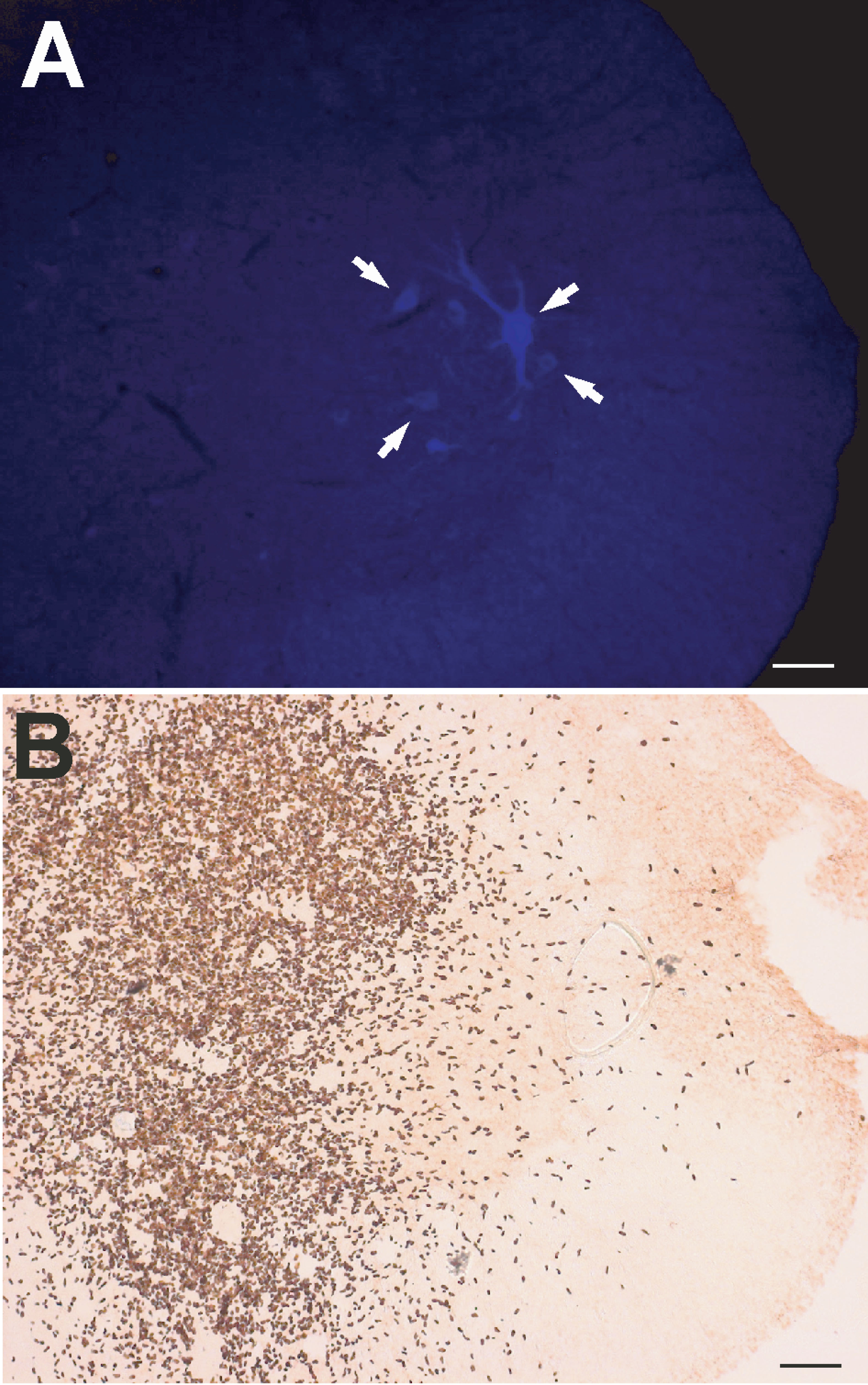

Supplement: Figure S2 — The correlation with the tracer labeled motor neurons and hNUC staining. hNPC were unilaterally transplanted into the lumbar spinal cord of wild type rats. After surgery, a retrograde tracer True Blue (2.5%) was injected into the same side as the hNPC transplants of the tibialis anterior muscle. Four days after transplantation, the spinal cord was collected and checked for true blue positive neurons in the lumbar spinal cord (A). Furthermore, the adjacent section was immunostained with hNUC antibody (B). Scale bars: 100 µm. (1.74 MB JPG) [file pone.0000689.s002.jpg]
